# Bringing Psychological Strategies to Robot-Assisted Physiotherapy for Enhanced Treatment Efficacy

**DOI:** 10.3389/fnins.2019.00984

**Published:** 2019-09-18

**Authors:** Bin Zhong, Wenxin Niu, Elizabeth Broadbent, Andrew McDaid, Tatia M. C. Lee, Mingming Zhang

**Affiliations:** ^1^Department of Biomedical Engineering, Southern University of Science and Technology, Shenzhen, China; ^2^Shanghai Yangzhi Rehabilitation Hospital, Tongji University School of Medicine, Shanghai, China; ^3^School of Medicine, The University of Auckland, Auckland, New Zealand; ^4^Department of Mechanical Engineering, The University of Auckland, Auckland, New Zealand; ^5^The State Key Laboratory of Brain and Cognitive Sciences, The University of Hong Kong, Hong Kong, China; ^6^Key Lab of Digital Manufacturing Equipment and Technology, Huazhong University of Science and Technology, Wuhan, China

**Keywords:** psychology, robot-assisted, physiotherapy, robotics, enhanced effect

## Abstract

Robotic technologies offer a range of functions to augment clinical rehabilitation practice. However, compliance with robot-assisted rehabilitation techniques has not been optimally achieved. Traditional approaches to improving the treatment efficacy are focusing more on the system function, while psychological factors have not been integrated comprehensively. In this perspective paper, eight key factors reflecting three conceptions-robot design, function design, and patients’ expectations have been evaluated and analyzed. Clinical results with 28 therapists and 84 patients indicate that integrating psychological strategies into robot-assisted physiotherapy may promote better trust and acceptance of rehabilitation robots.

## INTRODUCTION

Robotic technologies offer a range of functions to augment clinical rehabilitation practice. The Lokomat exoskeleton (Hidler et al., 2009), an established intervention for improving people’s walking ability, aids lower limb movement. The MIT-MANUS helps to retrain motor movement for human arms (Masia et al., 2007). A compliant parallel robot with the bioinspired design is able to deliver multiple ankle movements in three-dimensional space (Zhang et al., 2017, 2019). The Hocoma Armeo Spring was proved to be effective to enhance the upper-limb rehabilitation performance for high-level disability multiple sclerosis patients (Gijbels et al., 2011). A variety of assistive control strategies have also been proposed to maximize engagement from patients, aiming to promote neural plasticity and enhance treatment efficacy (Pehlivan et al., 2016; Li et al., 2017). While this has been actively researched throughout the world, compliance with robot-assisted rehabilitation techniques has not been optimally achieved.

Physical disabilities place a substantial physical and mental burden on patients and their families, which may predispose them to depression. Shreds of evidence show that physical independence leads to better cognition and mood (Barker-Collo, 2007), and in reverse effective treatment of post-injury depression positively affects rehabilitation outcomes (Lenzi et al., 2008). Active biomechanical and mental engagement of patients in physical therapy can be an important factor in successful rehabilitation. Integrating psychological strategies with robot-assisted physiotherapy is thus expected to enhance human–robot engagement and treatment outcomes. The theory behind is that using psychological strategies can help to increase patients’ positive attitudes towards rehabilitation robotics, which accordingly will increase acceptance to prescribed training.

One of the most pressing demands of the day is to promote patients’ trust in rehabilitation robotic systems that predispose their willingness to accept robot-prescribed training. A meta-analysis suggests that robot performance (e.g., reliability and failure rate) and attributes (e.g., proximity, robot personality, and anthropomorphism) are significant contributors to the development of trust in human–robot interaction (Hancock et al., 2011). Most recently, Kellmeyer et al. (2018) advocated paying attention to creating and maintaining trust in human-robot interaction in designing social robots for rehabilitation purposes.

This paper aims to appeal to researchers within the rehabilitation robotics community that immediate and special attention should be given to using psychological strategies to improve human-robot interaction and trust, in developing robot-assisted techniques for physical therapy.

## Proposed Psychological Strategies

Psychological features affect rehabilitative outcomes of patients involved in robotic treatment more than those in conventional rehabilitation (Bragoni et al., 2013). The aim of introducing psychological approaches to robotic physiotherapy is to improve patients’ trust of rehabilitation robots and thus enhance treatment efficacy. The basic principle is to encourage active biomechanical and mental engagement of patients in robot-assisted training (Maclean and Pound, 2000; Lotze et al., 2003). This requires roboticists to work with psychologists, physiotherapists, and domain experts in the relevant applications.

**FIGURE 1:**
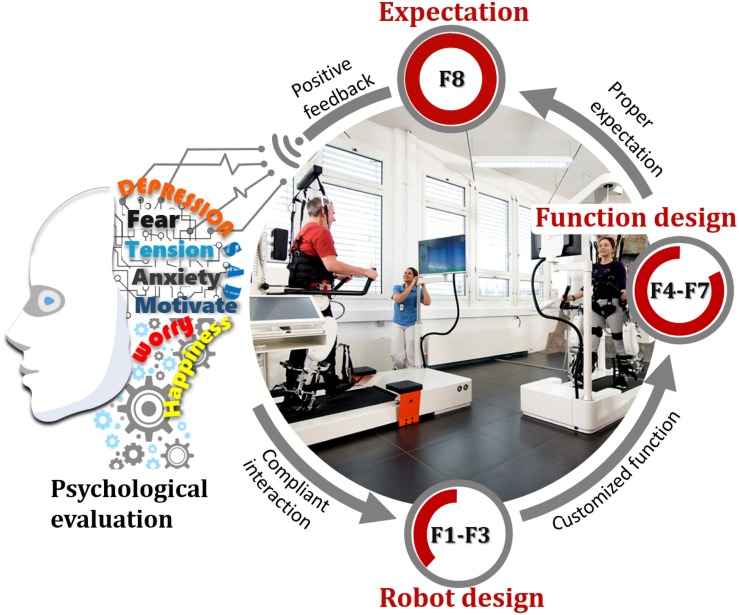
Proposed key psychological strategies to enhance robot-assisted therapy. Picture: Hocoma, Switzerland.

Eight factors (numbered as F1 to F8) including: F1-natural and compatible human-robot movement, F2-friendly robotic appearance, F3-attractive interface, F4-adaptable task difficulty levels, F5-intelligent conversation, F6-connecting individuals, F7- performance feedback, F8-accurate expectation, were proposed to enhance patients’ acceptance and trust on rehabilitation robots from the perspective of psychology, as in Figure 1. These eight factors fall in the group of robot design (F1 to F3), function design (F4 to F7), and patients’ expectation (F8), respectively. It is worth noting that the factors F1 to F8 are just part of potential psychological strategies, and more measures are waiting to be put forward and should be further explored.

Considering robot design, rehabilitation robotic systems should have natural and compatible movement with human users, a friendly appearance, and an attractive interface (Colombo et al., 2007). One way to achieve natural human–robot interaction is to use adaptive mechanisms that avoid joint misalignment to improve training safety and comfort. The appearance of robots is also critical since this provides cues about their abilities and propensities. An appropriate match between robots’ social cues and tasks can improve people’s acceptance of and cooperation with the robots. An attractive human-robot interface with virtual reality also contributes to maintaining patients’ interest in conducting repetitive training tasks.

In control design, affective computing can be taken to enhance physical and mental engagement. Affective computing integrates psychology, cognitive science, neuroscience, value-centered design, and ethics into engineering and computer science to address human needs. Emotion is fundamental to human survival and functioning, influencing cognition, perception, and activities of daily living. However, technologists have largely ignored emotion and created an often-frustrating experience for people, in part because emotion is intangible and patients’ mental states are difficult to be measured. Developing new technologies that incorporate affect theories to improve human engagement is critical. First, motor learning theory indicates that the learning rate is maximal at a task difficulty level that positively challenges and excites subjects while not being too stressful or boring (Guadagnoli and Lee, 2004). Task-difficulty adaptation to patients’ real-time psychological state improves rehabilitation results by challenging patients at an appropriate level (Koenig et al., 2011). Proper selection and processing of biological signals should also be considered in measuring people’s mental state with the robotic system. Second, an emotionally intelligent controller by adding natural conversation between human users and robots based on machine learning algorithms will greatly help to improve people’s mental status, especially by responding to a person’s frustration in a way that reduces negative feelings. Third, as a pioneering research direction, connecting individuals during robot-assisted training within the hospital or the community contributes to improving their self-awareness, affective state, and effective communication with others. Note that connecting individuals especially refers to bridging various robotic systems where many participants can conduct training tasks cooperatively or competitively. Last, providing or not immediate training performance feedback may also affect human users’ attitudes towards robotic therapy.

Matching individual expectations with robot abilities is another way to improve people’s psychological state. When robots have limitations in their abilities due to the development lag of science and technology, trust and acceptance can be increased by modifying human users’ expectations (Broadbent et al., 2009). The basic principle is to prepare patients with good information about the robot and the expected treatment efficacy. A mismatch can lead to inefficient teamwork, weakened trust, and even negative psychological effects.

## Pilot Study and Results

**FIGURE 2:**
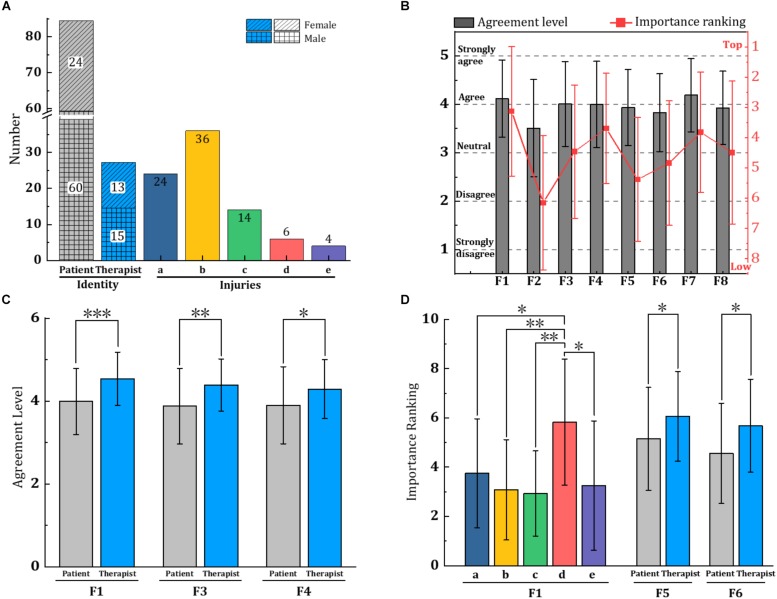
Statistical results from 28 therapists (age 25.4 ± 2.67 years old, height 167.4 ± 5.85 cm, weight 62.8 ± 13.3 kg) and 84 patients (age 42.1 ± 13.7 years old, height 166.02 ± 18.47 cm, weight 70.1 ± 21.6 kg), with ethics approval from Shanghai Sunshine Rehabilitation Center (2018083101). All participants have over-5-hours experience in robot-assisted therapy. In the charts, “a” to “e” denotes five types of injuries: a-musculoskeletal injury, b-stroke, c-spinal cord injury, d-brain trauma, and e-others (such as anthracaemia and brain tumor) in corresponding colors. **(A)** Describes the proportions of involved subjects and injuries; **(B)** illustrates mean values of the agreement level and importance ranking of eight factors from all 112 participants; **(C,D)** illustrate the ANOVA analysis results of identity and injuries over different factors. Asterisk denotes the significant difference between two elements, especially, ^∗∗∗^*P*-value < 0.001, ^∗∗^*P*-value < 0.01, and ^∗^*P*-value < 0.05.

An observational pilot study with 28 therapists and 84 patients with physical disabilities was conducted to preliminarily evaluate the impact of factors from F1 to F8. A questionnaire, as shown in Supplementary Data Sheet 2, was designed to investigate human user’s opinions about the influence of psychological strategies on robot-assisted physiotherapy. Before data collection, the investigators had a detailed presentation to 28 included therapists, and then each included therapist explained the questionnaire to each included patient, to ensure each participant well understand the questionnaire. As illustrated in Figure 2, the agreement level to each factor in the questionnaire is divided into five different levels, from “strongly disagree” to “strongly agree,” corresponding to “1” to “5” points, respectively. All the 112 subjects were required to answer every question with the corresponding point after careful consideration and rank the importance of these 8 factors at last from the top (rank 1) to low (rank 8) according to their feelings and using experience.

This study was approved by the Ethics Committee of Shanghai Sunshine Rehabilitation Center (2018083101), and written informed consent had been obtained from all the participants prior to conducting any experiments. All therapist participants are from the Sunshine Rehabilitation Center (Shanghai, China), with ages over 17 years old and over-five-hours experience in robot-assisted therapy. Patient participants can follow therapists’ instruction and understand the questionnaire well, with ages over 16 years old and over-five-hours experience in receiving robot-assisted therapy. Note that, the Sunshine Rehabilitation Center has the following robots, not limited to: (1) Hocoma^®^ Lokomat; (2) NeuroCom^®^ Balance Master; (3) Isomed 2000 System; (4) Imoove 600 System; (5) Reo^TM^ Ambulator; (6) Hocoma Armeo Spring; (7) AMADEO; (8) DIEGO; (9) Rehawalk^®^ System; (10) MOTOmed loop.la; (11) Pablo^®^ Upper Extremity; (12) *Saebo*ReJoyce; (13) E-LINK Evaluation and Exercise System. All participants have experience with operating or using these robotic devices.

Figure 2A illustrates the proportions of involved subjects and injuries. As in Figure 2B, results showed that all participants agreed with F1 to F8 in developing robotic therapy technology by considering psychological strategies, with rank of F7 > F1 > F3 > F4 > F5 > F8 > F6 > F2 in the agreement level, and rank of F1 > F4 > F7 > F3 > F8 > F6 > F5 > F2 in the importance ranking, top four factors are the same in both evaluation methods. Not surprisingly, natural and compatible human-robot movement (F1) and performance feedback (F7) were most valued. While a friendly appearance (F2) is less important.

Besides, a statistical one-way ANOVA analysis was carried out to investigate the effects of three different variables (the identity of participants, gender, and type of injury) on the evaluation of these eight factors. As illustrated in Figure 2C, there exists significant differences between patients and physiotherapists in agreement levels of F1 (*p <* 0.001), F3 (*p <* 0.01), and F4 (*p <* 0.05). Moreover, the effect of identity on importance ranking of F5 (*p <* 0.05) and F6 (*p <* 0.05) are also significant. Furthermore, therapists rated these five factors as more important than patients did. Figure 2D shows that type of injury was associated with a significant difference in ranking F1, patients with brain trauma rated natural and compatible human-robot movement (F1) as less importance during the interaction with robotic rehabilitation, it’s worth noting that the majority of these patients ranked performance feedback (F7) top among all factors, which can be obtained in the Supplementary Data Sheet 1. Nonetheless, gender makes no significant difference in both agreement level and importance ranking of F1 to F8 in this study, although gender usually leads to different perceptions in some rehabilitation therapy (Kakkad and Rathod, 2018).

In all, eight factors corresponding to rehabilitation robotics design had been preliminarily analyzed and the effects of three variables on the evaluation of these factors had also been investigated. The results demonstrated that the compatible human-robot movement and performance feedback should be promised, and other psychological strategies listed were also recognized to be effective to improve the rehabilitation performance. These results would contribute to the future design of human-robot rehabilitation system for peers in the rehabilitation robotics community to improve the human-robot interaction and rehabilitation efficacy.

## Discussion and Conclusion

This study proposed eight psychological strategies (F1 to F8) that may promote better trust and acceptance of rehabilitation robots by human users. It is essential to consider these factors in developing new robot-assisted rehabilitation techniques, especially in mechanical design, functional design, and the training protocol design. Preliminary data collected from 112 human users have positively supported the proposal of bringing psychological strategies into robot-assisted physiotherapy.

Note that, two main limitations exist in this study: (1) One is the small sample of therapist and patient participants. This may have made the statistical results of importance ranking not highly convincing as that presented in Figure 2, but the data on agreement levels are quite solid to advocate improving robot-assisted physiotherapy from the perspective of psychology. The other is that the proposed factors of F1 to F8 are just part of psychological considerations. It is obvious that more measures exist and are waiting to be proposed. (2) In addition, while the results may be dependent on geographic and demographic data, these findings of Chinese perceptions offer the potential to integrate psychological strategies into robot-assisted physiotherapy. Also, these psychological factors are not mutually exclusive, but we must understand their potential and benefits to maximize recovery and begin building a scientific basis for optimal robot-assisted rehabilitation practice.

In general, previous researches on rehabilitation robots (Lokomat, MIT-MANUS, Hocoma Armeo Spring, etc.) have laid a solid foundation for basic function implementation, and tremendous effort had been spent on improving reliable automation and the panoply of robots, while very little focus has been devoted to optimizing current robot-assisted physiotherapy from the viewpoint of psychology, for example, the factors F4, F5, F7, and F8. Literatures (Hancock et al., 2011; Kellmeyer et al., 2018) have evaluated the effects of human, robot, and environmental factors on perceived trust in human-robot interaction, while the factors F7 and F8 were not involved. Our results reveal that the compatible human-robot movement (F1) and performance feedback (F7), are the largest current effects on better human robot interaction. Comprehensively reexamining robot mechanism/control and training protocol design while considering patients’ psychological state deserves immediate attention. Future work should investigate specific mechanisms by which human users maintain positive attitudes towards robot-assisted physiotherapy and target these factors for intervention to maximize the positive outcomes of rehabilitation, and obtained findings are central to the consideration of coming rehabilitation robot design.

## Data Availability

The datasets analyzed in this manuscript are not publicly available. Requests to access the datasets should be directed to zhangmm@sustech.edu.cn.

## Ethics Statement

The studies involving human participants were reviewed and approved by this study was approved by the Ethics Committee of Shanghai Sunshine Rehabilitation Center (2018083101), and written informed consent had been obtained from all the participants prior to conducting any experiments. All therapist participants are from the Sunshine Rehabilitation Center (Shanghai, China), with ages over 17 years old and over-five-hours experience in robot-assisted therapy. Written informed consent to participate in this study was provided by the participants’ legal guardian/next of kin.

## Author Contributions

All authors listed have made a substantial, direct and intellectual contribution to the work, and approved it for publication.

## Conflict of Interest Statement

The authors declare that the research was conducted in the absence of any commercial or financial relationships that could be construed as a potential conflict of interest.

## References

[B1] Barker-Collo S. L. (2007). Depression and anxiety 3 months post stroke: prevalence and correlates. *Arch. Clin. Neuropsychol.* 22 519–531. 10.1016/j.acn.2007.03.002 17462857

[B2] Bragoni M. Broccoli M. Iosa M. Morone G. De Angelis D. Venturiero V. (2013). Influence of psychologic features on rehabilitation outcomes in patients with subacute stroke trained with robotic-aided walking therapy. *Am. J. Phys. Med. Rehabil.* 92 e16–e25. 10.1097/PHM.0b013e3182a20a34 24052026

[B3] Broadbent E. Stafford R. MacDonald B. (2009). Acceptance of healthcare robots for the older population: review and future directions. *Int. J. Soc. Robot.* 1 319–330. 10.1007/s12369-009-0030-6

[B4] Colombo R. Pisano F. Mazzone A. Delconte C. Micera S. Carrozza M. C. (2007). Design strategies to improve patient motivation during robot-aided rehabilitation. *J. Neuroeng. Rehabil.* 4:3. 17309790 10.1186/1743-0003-4-3 PMC1805445

[B5] Gijbels D. Lamers I. Kerkhofs L. Alders G. Knippenberg E. Feys P. (2011). The armeo spring as training tool to improve upper limb functionality in multiple sclerosis: a pilot study. *J. Neuroeng. Rehabil.* 8:5. 10.1186/1743-0003-8-5 21261965 PMC3037310

[B6] Guadagnoli M. A. Lee T. D. (2004). Challenge point: a framework for conceptualizing the effects of various practice conditions in motor learning. *J. Mot. Behav.* 36 212–224. 10.3200/jmbr.36.2.212-224 15130871

[B7] Hancock P. A. Billings D. R. Schaefer K. E. Chen J. Y. De Visser E. J. Parasuraman R. (2011). A meta-analysis of factors affecting trust in human-robot interaction. *Hum. Factors* 53 517–527. 10.1177/0018720811417254 22046724

[B8] Hidler J. Nichols D. Pelliccio M. Brady K. Campbell D. D. Kahn J. H. (2009). Multicenter randomized clinical trial evaluating the effectiveness of the lokomat in subacute stroke. *Neurorehabil. Neural Repair* 23 5–13. 10.1177/1545968308326632 19109447

[B9] Kakkad A. Rathod P. V. (2018). Factors affecting recovery after stroke: a narrative review. *Indian J. Physiother. Occup. Ther.* 12 22–27. 10.1016/j.bbr.2016.08.029 27531500 PMC5305670

[B10] Kellmeyer P. Mueller O. Feingold-Polak R. Levy-Tzedek S. (2018). Social robots in rehabilitation: a question of trust. *Sci. Robot.* 3:eaat1587 10.1126/scirobotics.aat1587 33141717

[B11] Koenig A. Omlin X. Zimmerli L. Sapa M. Krewer C. Bolliger M. (2011). Psychological state estimation from physiological recordings during robot-assisted gait rehabilitation. *J. Rehabil. Res. Dev.* 48 367–385. 21674389 10.1682/jrrd.2010.03.0044

[B12] Lenzi G. Altieri M. Maestrini I. (2008). Post-stroke depression. *Rev. Neurol.* 164 837–840. 10.1016/j.neurol.2008.07.010 18771785

[B13] Li X. Pan Y. Chen G. Yu H. (2017). Multi-modal control scheme for rehabilitation robotic exoskeletons. *Int. J. Robot. Res.* 36 759–777. 10.1177/0278364917691111

[B14] Lotze M. Braun C. Birbaumer N. Anders S. Cohen L. G. (2003). Motor learning elicited by voluntary drive. *Brain* 126 866–872. 10.1093/brain/awg079 12615644

[B15] Maclean N. Pound P. (2000). A critical review of the concept of patient motivation in the literature on physical rehabilitation. *Soc. Sci. Med.* 50 495–506. 10641802 10.1016/s0277-9536(99)00334-2

[B16] Masia L. Krebs H. I. Cappa P. Hogan N. (2007). Design and characterization of hand module for whole-arm rehabilitation following stroke. *IEEE ASME Trans. Mechatron.* 12 399–407. 10.1109/tmech.2007.901928 20228969 PMC2836734

[B17] Pehlivan A. U. Losey D. P. Malley M. K. O. (2016). Minimal assist-as-needed controller for upper limb robotic rehabilitation. *IEEE Trans. Robot.* 32 113–124. 10.1109/tro.2015.2503726

[B18] Zhang M. McDaid A. Veale A. J. Peng Y. Xie S. Q. (2019). Adaptive Trajectory tracking control of a parallel ankle rehabilitation robot with joint-space force distribution. *IEEE Access* 7 85812–85820. 10.1109/access.2019.2925182

[B19] Zhang M. Xie S. Q. Li X. Zhu G. Meng W. Huang X. (2017). Adaptive patient-cooperative control of a compliant ankle rehabilitation robot (CARR) with enhanced Training safety. *IEEE Trans. Ind. Electron.* 65 1398–1407. 10.1109/tie.2017.2733425

