# Supplementary material for: Bringing Psychological Strategies to Robot-Assisted Physiotherapy for Enhanced Treatment Efficacy
Source: Front Neurosci. 2019 Sep 18;13:984. doi: 10.3389/fnins.2019.00984 (PMC6759573; doi:10.3389/fnins.2019.00984)
Supplement: Supplementary file 1 [file Data_Sheet_1.pdf]

Data acquisition and analysis

1. General information of therapists and their questionnaire results

Note:
F1: Natural and compatible movement, F2: Friendly appearance, F3: Attractive interface, F4: Adaptable task levels, F5: Intelligent conversation, F6: Connecting individuals, F7: Performance feedback, F8: Accurate expectation.

Agreement level will be divided into five levels from strongly disagree to strongly agree corresponding to mark 1 to 5. In the importance ranking, number 1and 8 denote the top and last consideration respectively .

Number	Gender	Age	Height (cm)	Weight (kg)	Using time (h)
1	Male	25	165	65	10 to 20
2	Male	23	171	85	10 to 20
3	Male	25	165	80	10 to 20
4	Male	28	170	55	over 30
5	Male	25	175	90	over 30
6	Male	25	170	68	10 to 20
7	Female	24	170	56	over 30
8	Male	29	172	75	over 30
9	Male	27	174	58	over 30
10	Female	26	168	50	over 30
11	Male	25	173	63	over 30
12	Female	28	163	50	over 30
13	Female	29	175	65	over 30
14	Male	31	173	85	over 30
15	Male	26	160	60	over 30
16	Female	20	155	46	5 to 10
17	Female	25	157	50	over 30
18	Female	26	167	64	over 30
19	Male	22	170	59	over 30
20	Female	24	160	49	over 30
21	Male	31	178	92	over 30
22	Female	24	160	45	over 30
23	Female	23	162	50	5 to 10
24	Female	24	165	64	over 30
25	Female	22	164	53	5 to 10
26	Male	22	171	65	20 to 30
27	Male	27	165	57	over 30
28	Female	26	170	60	over 30

Results of agreement level							
F1	F2	F3	F4	F5	F6	F7	F8
5	5	5	5	5	5	5	5
4	4	4	3	4	4	4	4
3	4	4	4	3	4	4	3
5	4	5	5	4	3	4	5
5	3	4	4	5	4	5	4
3	4	3	5	4	5	5	5
4	4	5	4	3	5	4	4
5	4	5	5	4	5	5	4
4	4	4	4	4	3	4	4
4	4	3	4	4	2	4	4
5	3	5	4	4	3	5	4
4	4	4	4	4	4	4	4
4	5	5	4	4	3	4	4
5	4	5	4	4	4	5	3
5	4	5	5	5	5	4	5
5	4	4	4	4	4	4	5
4	4	4	5	5	4	5	4
5	4	4	5	5	5	4	5
5	4	5	5	4	4	4	4
4	4	4	4	4	3	4	4
4	4	5	5	5	4	5	4
5	3	5	5	4	3	3	5
5	4	4	4	4	4	4	4
5	2	4	4	4	4	3	4
5	3	5	4	4	4	3	3
5	4	4	5	5	4	5	4
5	2	4	2	3	3	5	3
5	3	5	4	4	5	5	4

Results of importance ranking							
F1	F2	F3	F4	F5	F6	F7	F8
4	3	1	5	2	6	7	8
1	4	7	6	8	3	5	2
1	8	4	2	7	5	3	6
7	3	6	5	8	1	4	2
1	8	3	2	5	4	7	6
1	2	3	4	5	6	7	8
2	7	6	3	8	5	1	4
1	6	2	4	8	5	3	7
5	4	6	1	8	7	2	3
4	6	5	3	7	8	1	2
1	8	4	6	7	3	5	2
1	8	5	4	6	7	2	3
1	6	4	3	7	8	5	2
1	4	3	5	6	7	2	8
6	7	8	1	5	2	3	4
3	4	7	2	7	5	1	6
3	2	1	5	4	7	6	8
2	3	1	5	4	8	7	6
1	8	7	2	4	6	3	5
3	8	5	2	7	5	4	1
3	1	2	4	5	8	6	7
2	7	4	3	8	6	5	1
1	8	7	3	2	4	6	5
2	8	7	1	6	5	3	4
1	4	2	3	4	7	8	6
1	4	2	3	8	7	5	6
1	5	4	3	6	8	2	7
1	7	5	4	8	6	2	3

2. General information of patients and their questionaire results

Note:

F1: Natural and compatible movement, F2: Friendly appearance, F3: Attractive interface, F4: Adaptable task levels, F5: Intelligent conversation, F6: Connecting individuals, F7: Performance feedback, F8: Accurate expectation.

Agreement level will be divided into five levels from strongly disagree to strongly agree corresponding to mark 1 to 5.
In the importance ranking, number 1and 8 denote the top and last consideration respectively .

Number	Gender	Age	Height (cm)	Weight (kg)	Using time (h)	Resutls of agreement level								Resutls of importance ranking							
						F1	F2	F3	F4	F5	F6	F7	F8	F1	F2	F3	F4	F5	F6	F7	F8
1	Male	64	155	60	10 to 20	5	4	5	5	5	5	5	5	4	3	2	5	6	1	7	8
2	Female	36	150	80	over 30	5	5	5	5	5	4	4	4	3	2	1	4	6	5	7	8
3	Male	69	170	80	5 to 10	5	5	5	5	5	5	5	5	3	2	1	4	5	6	7	8
4	Male	63	180	75	over 30	5	5	4	5	5	5	5	5	3	2	1	4	7	5	8	6
5	Female	65	157	64	20 to 30	4	2	4	4	4	4	4	4	3	8	7	1	6	5	2	4
6	Male	29	175	65	over 30	4	4	4	4	4	4	5	4	6	8	2	5	7	4	3	1
7	Female	26	152	36	5 to 10	4	3	3	4	4	4	4	4	4	8	7	1	6	5	3	2
8	Male	57	170	70	10 to 20	4	3	4	4	4	4	4	4	1	8	3	2	5	6	4	7
9	Female	33	160	55	over 30	4	5	5	5	4	5	5	5	3	2	1	4	5	7	6	8
10	Female	33	160	45	10 to 20	4	3	3	4	4	3	4	4	4	8	7	2	6	5	3	1
11	Male	55	175	74	over 30	3	4	4	4	4	4	2	4	7	8	4	6	3	5	1	2
12	Male	46	175	61	5 to 10	5	5	5	5	5	5	5	1	5	8	1	6	3	4	7	2
13	Male	30	168	83	over 30	4	2	4	3	1	5	5	4	2	7	8	4	6	3	1	5
14	Female	31	155	40	20 to 30	4	4	4	3	4	4	4	4	4	8	7	3	6	1	2	5
15	Male	31	168	60	over 30	4	3	3	3	4	4	3	3	8	7	5	3	6	4	1	2
16	Female	51	164	58	10 to 20	4	5	5	5	5	5	5	5	4	7	1	8	6	2	3	5
17	Male	40	170	55	over 30	4	4	4	4	4	4	4	4	1	8	5	2	7	6	4	3
18	Male	21	160	66	5 to 10	4	3	4	4	3	3	4	4	2	8	6	1	7	5	3	4
19	Male	62	174	73	10 to 20	4	4	4	4	4	4	4	4	2	1	6	4	5	3	8	7
20	Male	17	170	51	20 to 30	5	4	4	5	3	4	5	4	1	4	5	2	8	6	3	7
21	Male	30	175	67	5 to 10	4	4	4	4	4	4	4	4	1	2	3	4	5	6	7	8
22	Male	60	165	65	10 to 20	4	2	2	4	4	4	5	4	3	8	7	6	5	2	1	4
23	Male	26	175	61	over 30	5	2	4	4	5	4	5	4	6	8	1	5	2	3	4	7
24	Male	59	158	70	10 to 20	3	4	5	5	5	4	5	3	8	7	4	1	2	5	6	3
25	Male	30	174	67	5 to 10	4	4	4	4	4	4	4	4	7	8	5	4	6	2	1	3
26	Male	19	168	79	20 to 30	4	3	3	4	4	4	4	5	5	8	6	3	2	1	4	7
27	Male	68	174	73	10 to 20	3	4	4	4	3	2	2	2	6	8	7	3	4	5	1	2
28	Male	51	175	75	5 to 10	5	2	2	5	5	2	5	5	1	8	3	2	6	7	4	5
29	Male	51	150	56	5 to 10	4	3	5	4	3	4	4	4	1	6	3	4	8	7	2	5
30	Female	46	155	60	20 to 30	4	5	5	4	4	4	5	4	3	5	4	8	7	6	2	1
31	Male	31	172	58	5 to 10	4	4	4	4	4	4	4	4	5	7	6	4	8	2	3	1
32	Male	46	172	70	over 30	4	4	3	4	4	4	4	3	1	4	5	2	8	3	6	7
33	Male	45	168	92	over 30	4	4	4	2	4	4	4	4	7	8	5	6	4	2	3	1
34	Male	36	175	86	5 to 10	5	5	2	2	2	4	4	4	1	3	7	6	2	5	4	8
35	Male	35	180	81	5 to 10	5	2	4	4	3	2	4	3	2	3	6	7	1	8	4	5
36	Male	29	178	80	20 to 30	5	5	4	5	5	5	5	5	4	3	8	1	6	2	5	7
37	Female	38	160	53	5 to 10	5	5	5	5	5	3	3	3	1	4	3	2	7	8	5	6
38	Male	45	166	64	10 to 20	4	4	4	3	3	3	4	3	2	8	4	5	1	3	6	7
39	Female	30	160	49	10 to 20	4	3	4	5	3	4	4	3	6	7	3	1	2	4	5	8
40	Female	48	155	62	5 to 10	4	5	3	4	4	3	3	4	7	8	5	6	1	2	3	4
41	Male	28	175	58	10 to 20	4	4	3	4	3	4	3	4	5	8	4	3	1	2	7	6
42	Male	45	170	65	10 to 20	3	2	4	3	3	3	3	3	1	7	8	6	5	2	3	4
43	Male	55	168	60	10 to 20	5	2	5	5	5	5	5	5	1	7	3	4	6	5	2	8
44	Female	56	164	59	10 to 20	4	3	4	3	4	4	3	4	1	3	4	2	5	6	7	8
45	Male	24	178	70	10 to 20	4	3	4	2	4	3	4	3	1	7	2	5	3	6	4	8
46	Male	42	172	85	over 30	4	4	5	4	4	4	5	5	3	7	4	5	6	8	1	2
47	Female	25	158	57	over 30	4	2	5	4	4	4	5	5	1	8	5	2	6	7	3	4
48	Female	29	160	50	10 to 20	4	4	4	4	4	3	4	4	5	8	4	3	7	6	2	1
49	Female	37	162	57	over 30	4	2	4	2	2	4	4	4	1	8	5	7	6	3	2	4
50	Male	23	170	62	5 to 10	4	2	4	4	4	4	4	4	1	4	7	8	5	6	3	2
51	Female	40	160	53	over 30	4	2	2	2	2	4	4	2	1	8	6	5	7	4	2	3
52	Male	29	175	68	10 to 20	4	4	4	4	4	3	3	3	1	8	2	3	6	7	5	4
53	Male	41	174	85	10 to 20	3	2	5	5	2	4	2	2	4	7	1	2	8	3	5	6
54	Male	49	165	60	5 to 10	3	4	4	4	4	4	4	3	7	6	5	2	1	3	4	8
55	Male	65	178	70	10 to 20	3	2	3	4	4	5	2	3	6	8	7	1	2	3	5	4
56	Female	53	157	57	over 30	4	3	2	2	3	3	4	3	4	8	6	3	7	5	2	1
57	Male	43	173	71	20 to 30	4	4	4	4	4	4	4	4	1	8	7	5	6	4	2	3
58	Male	51	175	80	10 to 20	5	5	5	5	5	5	5	5	1	8	7	5	6	4	2	3
59	Male	38	177	84	over 30	4	4	4	4	4	4	4	4	1	3	4	5	2	7	6	8
60	Female	66	165	65	over 30	4	2	4	4	4	4	4	4	6	8	7	5	3	2	1	4
61	Male	72	174	60	over 30	4	2	4	4	4	4	5	4	3	8	7	4	2	6	5	1
62	Male	37	170	76	over 30	3	2	1	4	4	4	5	4	1	8	7	4	6	3	5	2
63	Male	60	175	81	over 30	2	4	2	2	2	2	4	4	3	8	4	5	6	7	1	2
64	Male	48	184	89	10 to 20	4	5	5	5	2	4	5	4	5	7	6	1	8	4	2	3
65	Female	34	157	51	5 to 10	4	3	4	4	4	4	4	4	3	8	1	7	2	4	5	6
66	Male	44	174	74	20 to 30	1	2	5	5	4	4	4	4	7	8	6	5	4	3	1	2
67	Male	33	178	71	20 to 30	5	3	4	4	4	4	5	4	1	7	6	4	5	8	2	3
68	Female	48	161	62	5 to 10	5	3	4	5	4	3	5	4	1	7	6	2	5	8	3	4
69	Male	24	172	68	over 30	4	3	4	3	4	4	4	3	4	8	1	2	7	3	5	6
70	Female	39	156	59	10 to 20	2	3	4	2	4	4	4	4	7	8	1	6	3	4	5	2
71	Male	47	170	88	5 to 10	4	5	5	4	4	4	4	4	3	6	5	1	7	8	4	2
72	Female	45	155	57	5 to 10	4	2	3	4	4	3	4	4	1	8	7	2	5	4	3	6
73	Female	53	160	75	over 30	3	2	4	4	4	2	5	2	5	7	8	1	6	4	3	2
74	Female	29	155	45	10 to 20	4	4	4	4	4	4	4	4	3	8	1	2	7	4	5	6
75	Male	35	180	99	over 30	4	4	4	4	4	4	5	4	4	3	1	5	6	8	7	2
76	Male	65	167	63	over 30	4	2	4	2	4	2	5	4	1	8	2	4	5	7	3	6
77	Male	28	182	76	10 to 20	4	4	4	4	4	4	4	4	3	8	7	2	5	6	1	4
78	Male	34	171	78	5 to 10	4	4	3	4	3	4	3	5	8	7	6	5	4	3	2	1
79	Male	24	170	65	5 to 10	5	3	4	5	4	3	5	4	5	2	3	6	8	1	4	7
80	Male	31	172	67	20 to 30	4	4	4	4	4	4	4	4	5	4	3	7	8	1	6	2
81	Male	49	168	73	20 to 30	4	4	4	4	4	4	4	4	3	5	8	1	6	7	2	4
82	Male	28	170	47	5 to 10	4	4	4	4	4	4	4	4	6	5	1	2	8	7	3	4
83	Male	53	162	70	over 30	1	1	1	1	5	1	5	5	3	2	4	5	1	7	6	8
84	Male	55	168	64	over 30	5	4	4	4	5	4	4	4	6	7	5	4	8	2	3	1

Injuries included in this survey	Number	Proportion
Musculoskeletal injury	24	28%
Stroke	36	43%
Spinal cord injury	14	17%
Brain trauma	6	7%
Others	4	5%

* Others (such as anthracaemia and brain tumor)

3. Data sheet in SPSS analysis process

Values of different labels in SPSS

Type of injuries	Value
Musculoskeletal injury	1
Stroke	2
Spinal cord injury	3
Brain trauma	4
Others	5

Using time	Value
5 to 10 hours	1
10 to 20 hours	2
20 to 30 hours	3
over 30 hours	4

Gender	Value
Male	1
Female	2
**Identity**	**Value**
Therapist	1
Patient	2

Note: Quesion1 is for patients only, question2 is for therapsits only. Question 1 and 2 are described in the attached questionnaire

Basic information										Agreement level								Importance ranking							
**Number**	**Gender**	**Age**	**Height (cm)**	**Weight (kg)**	**Using time**	**Identity**	**Injuries**	**Question 1**	**Question 2**	**F1**	**F2**	**F3**	**F4**	**F5**	**F6**	**F7**	**F8**	**F1**	**F2**	**F3**	**F4**	**F5**	**F6**	**F7**	**F8**
1	1	25	165	65	2	1			5	5	5	5	5	5	5	5	5	4	3	1	5	2	6	7	8
2	1	23	171	85	2	1			4	4	4	4	3	4	4	4	4	1	4	7	6	8	3	5	2
3	1	25	165	80	2	1			3	3	4	4	4	3	4	4	3	1	8	4	2	7	5	3	6
4	1	28	170	55	4	1			4	5	4	5	5	4	3	4	5	7	3	6	5	8	1	4	2
5	1	25	175	90	4	1			4	5	3	4	4	5	4	5	4	1	8	3	2	5	4	7	6
6	1	25	170	68	2	1			4	3	4	3	5	4	5	5	5	1	2	3	4	5	6	7	8
7	2	24	170	56	4	1			5	4	4	5	4	3	5	4	4	2	7	6	3	8	5	1	4
8	1	29	172	75	4	1			4	5	4	5	5	4	5	5	4	1	6	2	4	8	5	3	7
9	1	27	174	58	4	1			4	4	4	4	4	4	3	4	4	5	4	6	1	8	7	2	3
10	2	26	168	50	4	1			4	4	4	3	4	4	2	4	4	4	6	5	3	7	8	1	2
11	1	25	173	63	4	1			4	5	3	5	4	4	3	5	4	1	8	4	6	7	3	5	2
12	2	28	163	50	4	1			5	4	4	4	4	4	4	4	4	1	8	5	4	6	7	2	3
13	2	29	175	65	4	1			4	4	5	5	4	4	3	4	4	1	6	4	3	7	8	5	2
14	1	31	173	85	4	1			4	5	4	5	4	4	4	5	3	1	4	3	5	6	7	2	8
15	1	26	160	60	4	1			5	5	4	5	5	5	5	4	5	6	7	8	1	5	2	3	4
16	2	20	155	46	1	1			4	5	4	4	4	4	4	4	5	3	4	7	2	7	5	1	6
17	2	25	157	50	4	1			5	4	4	4	5	5	4	5	4	3	2	1	5	4	7	6	8
18	2	26	167	64	4	1			4	5	4	4	5	5	5	4	5	2	3	1	5	4	8	7	6
19	1	22	170	59	4	1			4	5	4	5	5	4	4	4	4	1	8	7	2	4	6	3	5
20	2	24	160	49	4	1			4	4	4	4	4	4	3	4	4	3	8	5	2	7	5	4	1
21	1	31	178	92	4	1			5	4	4	5	5	5	4	5	4	3	1	2	4	5	8	6	7
22	2	24	160	45	4	1			5	5	3	5	5	4	3	3	5	2	7	4	3	8	6	5	1
23	2	23	162	50	1	1			4	5	4	4	4	4	4	4	4	1	8	7	3	2	4	6	5
24	2	24	165	64	4	1			4	5	2	4	4	4	4	3	4	2	8	7	1	6	5	3	4
25	2	22	164	53	1	1			4	5	3	5	4	4	4	3	3	1	4	2	3	4	7	8	6
24	1	22	171	65	3	1			4	5	4	4	5	5	4	5	4	1	4	2	3	8	7	5	6
27	1	27	165	57	4	1			2	5	2	4	2	3	3	5	3	1	5	4	3	6	8	2	7
28	2	26	170	60	4	1			5	5	3	5	4	4	5	5	4	1	7	5	4	8	6	2	3
29	1	64	155	60	2	2	2	4		5	4	5	5	5	5	5	5	4	3	2	5	6	1	7	8
30	2	36	150	80	4	2	2	4		5	5	5	5	5	4	4	4	3	2	1	4	6	5	7	8
31	1	69	170	80	1	2	2	5		5	5	5	5	5	5	5	5	3	2	1	4	5	6	7	8
32	1	63	180	75	4	2	2	4		5	5	4	5	5	5	5	5	3	2	1	4	7	5	8	6
33	2	65	157	64	3	2	2	2		4	2	4	4	4	4	4	4	3	8	7	1	6	5	2	4
34	1	29	175	65	4	2	5	4		4	4	4	4	4	4	5	4	6	8	2	5	7	4	3	1
35	2	26	152	36	1	2	3	4		4	3	3	4	4	4	4	4	4	8	7	1	6	5	3	2
36	1	57	170	70	2	2	3	3		4	3	4	4	4	4	4	4	1	8	3	2	5	6	4	7
37	2	33	160	55	4	2	3	4		4	5	5	5	4	5	5	5	3	2	1	4	5	7	6	8
38	2	33	160	45	2	2	3	4		4	3	3	4	4	3	4	4	4	8	7	2	6	5	3	1
39	1	55	175	74	4	2	4	3		3	4	4	4	4	4	2	4	7	8	4	6	3	5	1	2
40	1	46	175	61	1	2	2	2		5	5	5	5	5	5	5	1	5	8	1	6	3	4	7	2
41	1	30	168	83	4	2	3	1		4	2	4	3	1	5	5	4	2	7	8	4	6	3	1	5
42	2	31	155	40	3	2	3	4		4	4	4	3	4	4	4	4	4	8	7	3	6	1	2	5
43	1	31	168	60	4	2	4	4		4	3	3	3	4	4	3	3	8	7	5	3	6	4	1	2
44	2	51	164	58	2	2	2	4		4	5	5	5	5	5	5	5	4	7	1	8	6	2	3	5
45	1	40	170	55	4	2	2	4		4	4	4	4	4	4	4	4	1	8	5	2	7	6	4	3
46	1	21	160	66	1	2	3	3		4	3	4	4	3	3	4	4	2	8	6	1	7	5	3	4
47	1	62	174	73	2	2	2	4		4	4	4	4	4	4	4	4	2	1	6	4	5	3	8	7
48	1	17	170	51	3	2	3	4		5	4	4	5	3	4	5	4	1	4	5	2	8	6	3	7
49	1	30	175	67	1	2	3	4		4	4	4	4	4	4	4	4	1	2	3	4	5	6	7	8
50	1	60	165	65	2	2	2	2		4	2	2	4	4	4	5	4	3	8	7	6	5	2	1	4
51	1	26	175	61	4	2	2	5		5	2	4	4	5	4	5	4	6	8	1	5	2	3	4	7
52	1	59	158	70	2	2	2	4		3	4	5	5	5	4	5	3	8	7	4	1	2	5	6	3
53	1	30	174	67	1	2	4	4		4	4	4	4	4	4	4	4	7	8	5	4	6	2	1	3
54	1	19	168	79	3	2	2	5		4	3	3	4	4	4	4	5	5	8	6	3	2	1	4	7
55	1	68	174	73	2	2	2	3		3	4	4	4	3	2	2	2	6	8	7	3	4	5	1	2
56	1	51	175	75	1	2	2	2		5	2	2	5	5	2	5	5	1	8	3	2	6	7	4	5
57	1	51	150	56	1	2	1	2		4	3	5	4	3	4	4	4	1	6	3	4	8	7	2	5
58	2	46	155	60	3	2	1	4		4	5	5	4	4	4	5	4	3	5	4	8	7	6	2	1
59	1	31	172	58	1	2	1	4		4	4	4	4	4	4	4	4	5	7	6	4	8	2	3	1
60	1	46	172	70	4	2	2	4		4	4	3	4	4	4	4	3	1	4	5	2	8	3	6	7
61	1	45	168	92	4	2	4	4		4	4	4	2	4	4	4	4	7	8	5	6	4	2	3	1
62	1	36	175	86	1	2	2	5		5	5	2	2	2	4	4	4	1	3	7	6	2	5	4	8
63	1	35	180	81	1	2	2	5		5	2	4	4	3	2	4	3	2	3	6	7	1	8	4	5
64	1	29	178	80	3	2	1	5		5	5	4	5	5	5	5	5	4	3	8	1	6	2	5	7
65	2	38	160	53	1	2	1	4		5	5	5	5	5	3	3	3	1	4	3	2	7	8	5	6
66	1	45	166	64	2	2	1	4		4	4	4	3	3	3	4	3	2	8	4	5	1	3	6	7
67	2	30	160	49	2	2	1	4		4	3	4	5	3	4	4	3	6	7	3	1	2	4	5	8
68	2	48	155	62	1	2	1	2		4	5	3	4	4	3	3	4	7	8	5	6	1	2	3	4
69	1	28	175	58	2	2	1	3		4	4	3	4	3	4	3	4	5	8	4	3	1	2	7	6
70	1	45	170	65	2	2	2	3		3	2	4	3	3	3	3	3	1	7	8	6	5	2	3	4
71	1	55	168	60	2	2	2	3		5	2	5	5	5	5	5	5	1	7	3	4	6	5	2	8
72	2	56	164	59	2	2	2	3		4	3	4	3	4	4	3	4	1	3	4	2	5	6	7	8
73	1	24	178	70	2	2	3	4		4	3	4	2	4	3	4	3	1	7	2	5	3	6	4	8
74	1	42	172	85	4	2	2	4		4	4	5	4	4	4	5	5	3	7	4	5	6	8	1	2
75	2	25	158	57	4	2	4	4		4	2	5	4	4	4	5	5	1	8	5	2	6	7	3	4
76	2	29	160	50	2	2	4	4		4	4	4	4	4	3	4	4	5	8	4	3	7	6	2	1
77	2	37	162	57	4	2	1	2		4	2	4	2	2	4	4	4	1	8	5	7	6	3	2	4
78	1	23	170	62	1	2	1	2		4	2	4	4	4	4	4	4	1	4	7	8	5	6	3	2
79	2	40	160	53	4	2	5	3		4	2	2	2	2	4	4	2	1	8	6	5	7	4	2	3
80	1	29	175	68	2	2	5	5		4	4	4	4	4	3	3	3	1	8	2	3	6	7	5	4
81	1	41	174	85	2	2	2	4		3	2	5	5	2	4	2	2	4	7	1	2	8	3	5	6
82	1	49	165	60	1	2	1	4		3	4	4	4	4	4	4	3	7	6	5	2	1	3	4	8
83	1	65	178	70	2	2	2	3		3	2	3	4	4	5	2	3	6	8	7	1	2	3	5	4
84	2	53	157	57	4	2	2	4		4	3	2	2	3	3	4	3	4	8	6	3	7	5	2	1
85	1	43	173	71	3	2	2	4		4	4	4	4	4	4	4	4	1	8	7	5	6	4	2	3
86	1	51	175	80	2	2	2	5		5	5	5	5	5	5	5	5	1	8	7	5	6	4	2	3
87	1	38	177	84	4	2	2	4		4	4	4	4	4	4	4	4	1	3	4	5	2	7	6	8
88	2	66	165	65	4	2	2	2		4	2	4	4	4	4	4	4	6	8	7	5	3	2	1	4
89	1	72	174	60	4	2	2	4		4	2	4	4	4	4	5	4	3	8	7	4	2	6	5	1
90	1	37	170	76	4	2	2	4		3	2	1	4	4	4	5	4	1	8	7	4	6	3	5	2
91	1	60	175	81	4	2	2	2		2	4	2	2	2	2	4	4	3	8	4	5	6	7	1	2
92	1	48	184	89	2	2	2	5		4	5	5	5	2	4	5	4	5	7	6	1	8	4	2	3
93	2	34	157	51	1	2	1	4		4	3	4	4	4	4	4	4	3	8	1	7	2	4	5	6
96	1	44	174	74	3	2	2	1		1	2	5	5	4	4	4	4	7	8	6	5	4	3	1	2
94	1	33	178	71	3	2	2	1		5	3	4	4	4	4	5	4	1	7	6	4	5	8	2	3
95	2	48	161	62	1	2	1	1		5	3	4	5	4	3	5	4	1	7	6	2	5	8	3	4
97	1	24	172	68	4	2	1	4		4	3	4	3	4	4	4	3	4	8	1	2	7	3	5	6
98	2	39	156	59	2	2	1	4		2	3	4	2	4	4	4	4	7	8	1	6	3	4	5	2
99	1	47	170	88	1	2	1	4		4	5	5	4	4	4	4	4	3	6	5	1	7	8	4	2
100	2	45	155	57	1	2	1	2		4	2	3	4	4	3	4	4	1	8	7	2	5	4	3	6
101	2	53	160	75	4	2	1	1		3	2	4	4	4	2	5	2	5	7	8	1	6	4	3	2
102	2	29	155	45	2	2	1	2		4	4	4	4	4	4	4	4	3	8	1	2	7	4	5	6
103	1	35	180	99	4	2	1	2		4	4	4	4	4	4	5	4	4	3	1	5	6	8	7	2
104	1	65	167	63	4	2	2	2		4	2	4	2	4	2	5	4	1	8	2	4	5	7	3	6
105	1	28	182	76	2	2	1	3		4	4	4	4	4	4	4	4	3	8	7	2	5	6	1	4
106	1	34	171	78	1	2	1	4		4	4	3	4	3	4	3	5	8	7	6	5	4	3	2	1
107	1	24	170	65	1	2	1	3		5	3	4	5	4	3	5	4	5	2	3	6	8	1	4	7
108	1	31	172	67	3	2	5	4		4	4	4	4	4	4	4	4	5	4	3	7	8	1	6	2
109	1	49	168	73	3	2	3	4		4	4	4	4	4	4	4	4	3	5	8	1	6	7	2	4
110	1	28	170	47	1	2	3	4		4	4	4	4	4	4	4	4	6	5	1	2	8	7	3	4
111	1	53	162	70	4	2	3	1		1	1	1	1	5	1	5	5	3	2	4	5	1	7	6	8
112	1	55	168	64	4	2	3	2		5	4	4	4	5	4	4	4	6	7	5	4	8	2	3	1

4. Statistics analysis

Step 1: General Information

Identity		Age	Height (cm)	Weight (kg)
Therapists	Average	25.40	167.40	62.80
	Varience	2.67	5.85	13.34
Patients	Average	42.10	167.70	66.60
	Varience	13.68	8.21	12.20

Identity	Gender	Number
Therapists	Male	15
	Female	13
Patients	Male	60
	Female	24

Identity	Experience in using rehabilitation robots/Number of human			
	5-10 hours	10-20 hours	20-30 hours	over 30 hours
Therapists	3	4	1	20
Patients	22	24	11	27
Total	25	28	12	47

Injuries in this study	Number of patients	Proportion
Musculoskeletal injury	24	28%
Stroke	36	43%
Spinal cord injury	14	17%
Brain trauma	6	7%
Others	4	5%

Step2: Results of agreement level and importance ranking of participants

Agreement level									
		F1	F2	F3	F4	F5	F6	F7	F8
Therapists	Average	4.54	3.75	4.39	4.29	4.14	3.93	4.29	4.11
	Varience	0.64	0.70	0.63	0.71	0.59	0.81	0.66	0.63
	Ranking	F1>F3>F4=F7>F5>F8>F6>F2							
Patients	Average	3.99	3.43	3.88	3.90	3.87	3.80	4.15	3.87
	Varience	0.80	1.08	0.91	0.93	0.83	0.80	0.78	0.79
	Ranking	F7>F1>F4>F3>F8=F5>F6>F2							
Total	Average	4.13	3.51	4.01	4.00	3.94	3.83	4.19	3.93
	Varience	0.80	1.00	0.88	0.89	0.79	0.80	0.75	0.76
	Ranking	**F7>F1>F3>F4>F5>F8>F6>F2**							

Importance ranking									
		F1	F2	F3	F4	F5	F6	F7	F8
Therapists	Average	2.18	5.46	4.32	3.36	6.07	5.68	4.11	4.71
	Varience	1.68	2.25	2.11	1.45	1.82	1.89	2.11	2.31
	Ranking	F1>F4>F7>F3>F8>F2>F6>F5							
Patients	Average	3.45	6.38	4.51	3.80	5.15	4.56	3.73	4.42
	Varience	2.19	2.17	2.24	1.92	2.09	2.04	1.95	2.39
	Ranking	F1>F7>F4>F8>F3>F6>F5>F2							
Total	Average	3.13	6.15	4.46	3.69	5.38	4.84	3.82	4.49
	Varience	2.14	2.22	2.20	1.82	2.05	2.06	1.99	2.36
	Ranking	**F1>F4>F7>F3>F8>F6>F5>F2**							

Step3: One-way ANOVA analysis to investigate the significant difference

Note: Cells in gray and red indicate significant differences

ANOVA analysis results										
Variables		F1	F2	F3	F4	F5	F6	F7	F8	Significant differtence in agreement level
Injuries	H-test	0.113	0	0.027	0.676	0.096	0.601	0.166	0.148	
	ANOVA	0.062	0.683	0.105	0.12	0.472	0.918	0.483	0.287	
Identity	H-test	0.372	0	0.69	0.625	0.279	0.794	0.831	0.452	
	ANOVA	0.001	0.143	0.007	0.049	0.111	0.458	0.428	0.15	
Gender	H-test	0.148	0.518	0.958	0.735	0.113	0.708	0.02	0.349	
	ANOVA	0.551	0.716	0.703	0.823	0.937	0.499	0.189	0.865	

ANOVA analysis results										
Variables		F1	F2	F3	F4	F5	F6	F7	F8	Significant difference in importance ranking
Injuries	H-test	0.378	0.004	0.029	0.017	0.129	0.914	0.064	0.074	
	ANOVA	0.003	0.181	0.856	0.211	0.085	0.142	0.218	0.065	
Identity	H-test	0.024	0.479	0.526	0.053	0.461	0.298	0.404	0.791	
	ANOVA	0.006	0.058	0.694	0.27	0.04	0.012	0.382	0.566	
Gender	H-test	0.012	0.106	0.784	0.456	0.227	0.054	0.934	0.202	
	ANOVA	0.402	0.194	0.941	0.354	0.573	0.207	0.521	0.303	

Note: H-test: Homogeneity of variance test, **ANOVA**:One-way ANOVA analysis process.

Step4: Further investigation of the significant difference between each two types of injuries in ranking the importan

Note: Cells in red indicate significant differences

Injury i	Injury j	i-j mean difference	standard deviation	significance	Lower confidence of interval	pper confidence of interva
a	b	0.667	0.529	0.211	-0.38	1.72
	c	0.821	0.676	0.227	-0.52	2.16
	d	-2.083*	0.917	0.025	-3.9	-.27*
	e	0.5	1.085	0.646	-1.65	2.65
b	a	-0.667	0.529	0.211	-1.72	0.38
	c	0.155	0.633	0.807	-1.1	1.41
	d	-2.750*	0.886	0.002	-4.51	-.99*
	e	-0.167	1.059	0.875	-2.27	1.93
c	a	-0.821	0.676	0.227	-2.16	0.52
	b	-0.155	0.633	0.807	-1.41	1.1
	d	-2.905*	0.98	0.004	-4.85	-.96*
	e	-0.321	1.139	0.778	-2.58	1.94
d	a	2.083*	0.917	0.025	0.27	3.90*
	b	2.750*	0.886	0.002	0.99	4.51*
	c	2.905*	0.98	0.004	0.96	4.85*
	e	2.583*	1.297	0.049	0.01	5.15*
e	a	-0.5	1.085	0.646	-2.65	1.65
	b	0.167	1.059	0.875	-1.93	2.27
	c	0.321	1.139	0.778	-1.94	2.58
	d	-2.583*	1.297	0.049	-5.15	-.01*

Note: a: musculoskeletal injury, **b**: stroke, **c**: spinal cord injury, **d**: brain trauma, **e**: others

Importance ranking of F1 from 6 brain trauma patients

F1
2
1
2
2
8
4
