# Supplementary material for: Bringing Psychological Strategies to Robot-Assisted Physiotherapy for Enhanced Treatment Efficacy
Source: Front Neurosci. 2019 Sep 18;13:984. doi: 10.3389/fnins.2019.00984 (PMC6759573; doi:10.3389/fnins.2019.00984)
Supplement: Supplementary file 2 [file Data_Sheet_2.PDF]

Questionnaire

General information	Your name?	
	Gender	1: Male, 2: Female
	Age	
	Height	
	Weight	
	Accumulated time for robot-assisted rehabilitation training with robot assistance	1: 5-10 hours<br>2: 10-20 hours<br>3: 20-30 hours<br>4: over 30 hours
	Your identity?	1: Therapist, 2: Patient
	If you are a patient, which of the following injuries are you suffering from?<br>1: Musculoskeletal injury<br>2: Stroke<br>3: Spinal cord injury<br>4: Brain trauma<br>5: Others (such as anthracaemia and brain tumor)	
Patient only	Question 1: Mental state (especially the moods) can affect your willingness towards robot-assisted therapy.	1: Strongly disagree<br>2: Disagree<br>3: Neutral<br>4: Agree<br>5: Strongly agree
Therapist only	Question 2: A pre-evaluation of patients' psychological state is necessary in identifying appropriate candidates for robotic therapy.	1: Strongly disagree<br>2: Disagree<br>3: Neutral<br>4: Agree<br>5: Strongly agree

Robot design	**F1**: Natural and compatible human-robot movement will affect people's willingness to use and trust in robotic physiotherapy.<br><br>(Natural and compatible movement specially means that the robot joints are consistent with human body joints, or the has adaptive joint alignment ability.	1: Strongly disagree<br>2: Disagree<br>3: Neutral<br>4: Agree<br>5: Strongly agree
	**F2**: Friendly appearance of rehabilitation robots (critical to people's initial responses) will improve people's acceptance of and cooperation with the robot.	1: Strongly disagree<br>2: Disagree<br>3: Neutral<br>4: Agree<br>5: Strongly agree
	**F3**: An attractive interface with virtual reality will contribute to the maintenance of patients' interest in conducting repetitive training tasks with rehabilitation robots.	1: Strongly disagree<br>2: Disagree<br>3: Neutral<br>4: Agree<br>5: Strongly agree
Function design	**F4**: Task levels adaptive to the users' psychological state (such as stress and mood) can challenge patients at an appropriate level and avoid frustration.	1: Strongly disagree<br>2: Disagree<br>3: Neutral<br>4: Agree<br>5: Strongly agree
	**F5**: Intelligent robotic conversation will help to improve human users' mental status, especially responding to a person's frustration in a way that reduces negative feelings.	1: Strongly disagree<br>2: Disagree<br>3: Neutral<br>4: Agree<br>5: Strongly agree
	**F6**: Co-operation or competition with other individuals during robotic physiotherapy will contribute to improved self-awareness, affective state and effective communication with others.	1: Strongly disagree<br>2: Disagree<br>3: Neutral<br>4: Agree<br>5: Strongly agree
	**F7**: After robot-assisted rehabilitation training, it is necessary for the system to immediately present quantitative performance feedback to patients, helping them understand the training progress.	1: Strongly disagree<br>2: Disagree<br>3: Neutral<br>4: Agree

		5: Strongly agree	
Expectation	**F8**: Human users' accurate expectations about the capability/function of rehabilitation robots will help patients avoiding feelings of loss, inefficient man-machine cooperation, and weak trust.	1: Strongly disagree<br>2: Disagree<br>3: Neutral<br>4: Agree<br>5: Strongly agree	
Importance ranking	Order these eight psychological strategies (F1 to F8) in terms of importance.<br><br>Numbers “1 to 8” refers to the ranking of importance levels, where “1” is the most important one.	F1: Natural and compatible movement	
		F2: Friendly appearance	
		F3: Attractive interface	
		F4: Adaptable task levels	
		F5: Intelligent conversation	
		F6: Connecting individuals	
		F7: Performance feedback	
		F8: Accurate expectations	
Other psychological strategic factors you think are important?			
